# Evaluating a brief MBCT programme for non-suicidal self-injury in individuals with BPD: a within-subject pre–post pilot study

**DOI:** 10.1186/s40479-026-00337-3

**Published:** 2026-03-09

**Authors:** Szilvia Kresznerits, Ágnes Zinner-Gérecz, Mónika Miklósi, Tamás Szekeres, Dóra Perczel-Forintos

**Affiliations:** 1 Department of Clinical Psychology, Faculty of Medicine, Semmelweis University, Budapest, Hungary; 2 Mental Health Sciences Division, Doctoral School of Semmelweis University, Budapest, Hungary; 3 Department of Developmental and Clinical Child Psychology, Institute of Psychology, Eötvös Loránd University, Budapest, Hungary; 4 Centre of Mental Health, Pál Heim Children’s Hospital, Budapest, Hungary; 5 Department of Rehabilitation, National Institute of Oncology, Budapest, Hungary

**Keywords:** Borderline personality disorder, Self-mutilation, Mindfulness, Emotional regulation, Self-compassion, Suicide prevention

## Abstract

**Background:**

Non-suicidal self-injury (NSSI) is highly prevalent among individuals with borderline personality disorder (BPD) and a robust predictor of future suicide attempts and death. Access to comprehensive treatments such as dialectical behaviour therapy remains limited.

**Methods:**

This exploratory study evaluated the feasibility and preliminary outcomes of a brief, group-based mindfulness-based cognitive therapy program tailored to reduce NSSI (MBCT-NSSI) and improve associated psychological processes. Using a nonrandomised within-subject pre–post design with a waiting-period comparison, assessments were conducted at baseline (T1; *n* = 120), pre-intervention (T2; *n* = 72), and post-intervention (T3; *n* = 50). Outpatients with BPD and recent NSSI participated in a 9-week MBCT-NSSI group programme; key exclusions were acute suicide risk, psychosis, mania, and severe substance use. Primary outcomes were NSSI frequency and mindfulness. Secondary outcomes included self-compassion, self-esteem, impulsivity, depression, dissociation, and emotion regulation. Within-subject changes were analysed via linear mixed modelling and Wilcoxon signed-rank tests. Binary logistic regression identified predictors of dropout among eligible participants. No blinding or randomisation procedures were applied.

**Results:**

Among completers (*N* = 50), primary outcomes showed a significant reduction in NSSI frequency and an increase in mindfulness. Improvements were also observed across several secondary outcomes, including depressive symptoms, impulsivity, and emotion regulation. For mindfulness and secondary self-report outcomes, changes were observed between T2 and T3 but not during the waiting-list period. For NSSI, which was evaluated only at T1 and T3, findings should be interpreted as preliminary signal detection. Dissociative symptoms showed a non-significant trend toward improvement. Dropout among eligible patients was associated with higher impulsivity, maladaptive regulation, and, unexpectedly, higher self-compassion, whereas fewer comorbid diagnoses predicted lower treatment retention.

**Conclusions:**

MBCT-NSSI may be a feasible and potentially beneficial adjunctive intervention for individuals with BPD who engage in NSSI, with improvements in emotion regulation and preliminary signals of reduced self-harm frequency. These findings support further investigation through controlled trials and longer follow-up.

**Trial registration:**

Not applicable. The study had a within-subject pre–post design without a control condition, and therefore does not meet the ICMJE definition of a clinical trial. De-identified data are openly available via the Open Science Framework (OSF): 10.17605/OSF.IO/ZUR84.

**Supplementary Information:**

The online version contains supplementary material available at 10.1186/s40479-026-00337-3.

## Introduction

*“You can’t stop the waves*,* but you can learn to surf.”* This quote by Jon Kabat-Zinn not only captures the core philosophy of mindfulness but also reflects the lived experience of individuals with borderline personality disorder (BPD), whose lives are often shaped by emotional turbulence. BPD is a complex psychiatric condition characterised by enduring difficulties in emotion regulation, impulsivity, self-image, and interpersonal functioning. It is frequently associated with high rates of psychiatric comorbidity, functional impairment, and increased use of healthcare services [1–5].

Among the most concerning behaviours associated with BPD is non-suicidal self-injury (NSSI), defined as the deliberate infliction of harm to one’s own body without suicidal intent [6, 7]. NSSI is highly prevalent in this population and has multiple functions: it may act as a maladaptive coping strategy to alleviate emotional distress, a form of self-punishment, or a compulsive behaviour with addictive features—all of which are associated with elevated risk for future suicide attempts [8–12].

Despite the high level of clinical need, individuals with BPD often face substantial barriers to accessing evidence-based care. These include long waiting lists, clinician stigma, and limited availability of specialised treatments [13–18]. While comprehensive interventions such as Dialectical Behaviour Therapy (DBT) remain the gold standard treatments for BPD [19–21], their duration and complexity often limit their accessibility and scalability in real-world clinical settings.

Consequently, there has been a growing interest in low-intensity, time-limited psychotherapeutic interventions that can be more widely implemented, especially within outpatient settings [22, 23]. These interventions—often structured around brief group formats—have the potential to engage patients earlier in their treatment trajectory, reduce risk, and build foundational skills prior to or alongside longer-term therapies.

Emotion dysregulation has been consistently identified as a central mechanism underpinning both BPD symptoms and NSSI [20, 24–26]. Theoretical models implicate deficits in self-regulation, impulsivity, experiential avoidance, and low self-compassion in the onset and maintenance of NSSI [27–31]. According to the mindfulness deficit theory, individuals with BPD experience heightened impulsivity and emotion dysregulation due to reduced mindfulness capacity, resulting in greater reliance on maladaptive coping strategies such as substance use or self-injury [32–34]. Recent findings further support this model, with trait mindfulness shown to negatively correlate with BPD symptom severity [35], underscoring its relevance as a treatment target.

Mindfulness-based interventions (MBIs) aim to improve emotional functioning by cultivating present-moment awareness, non-judgmental acceptance, and behavioural flexibility [36, 37]. Mindfulness-based cognitive therapy (MBCT), originally developed to prevent relapse in depression, has been adapted for suicide risk populations [38, 39]. However, evidence remains limited for MBCT-format interventions specifically adapted to target ongoing NSSI in clinically diagnosed BPD samples, and available findings are largely drawn from broader MBIs or DBT-based mindfulness training [37, 40].

Emerging evidence suggests that brief mindfulness- and compassion-based interventions may be associated with improvements in NSSI-related outcomes and relevant psychological processes in non-clinical and adolescent samples [41–43]. Developing mindfulness skills is also a core component of DBT: mindfulness skills have been linked to improvements in impulsivity and to supporting self-injury inhibition through improved distress tolerance and behavioural inhibition [44–47]. In clinically diagnosed BPD samples, DBT-based mindfulness training has been associated with reductions in impulsivity and emotional reactivity, and neuroimaging findings suggest changes in default mode network functioning [40, 48, 49]. Moreover, interventions targeting self-compassion (e.g., loving-kindness meditation) may be relevant to shame-related processes and self-injurious behaviours [31, 49–51].

In addition, MBCT has been suggested as a potentially useful approach for NSSI. However, the most directly relevant published randomised trial protocol comparing group MBCT with group support in young people with NSSI [52] did not report outcome data and excluded individuals with a BPD diagnosis. Thus, outcome evidence remains limited for an MBCT-format group programme specifically adapted to address ongoing NSSI in clinically diagnosed BPD outpatients. The present pilot study addresses this gap by evaluating the feasibility and preliminary outcomes of a brief, adapted MBCT programme (MBCT-NSSI) in this population.

### Aims

This within-subject pre–post study aimed to evaluate the feasibility and preliminary outcomes of a 9-week MBCT-NSSI programme. The primary objective was to explore the feasibility and preliminary clinical outcomes of the MBCT-NSSI intervention, including potential changes in the frequency and severity of self-injury and related psychological mechanisms, such as mindfulness, emotion regulation, and impulsivity.

A secondary aim was to examine psychological predictors of dropout, thereby gaining a better understanding of the factors influencing group retention and engagement. Across psychotherapeutic interventions for BPD and other personality disorders, treatment dropout has emerged as a frequent and clinically significant challenge, particularly in group-based and mindfulness-oriented treatments, where emotional dysregulation, impulsivity, and interpersonal sensitivity may interfere with sustained engagement [53–55]. A growing body of evidence indicates that dropout is not random but systematically associated with baseline patient characteristics, including younger age, heightened emotional dysregulation, impulsivity, distress intolerance, trauma history, and weaker therapeutic alliance across DBT, MBT, schema therapy, and other structured treatments [53, 54, 56–58]. Previous studies have reported elevated dropout rates in structured psychotherapeutic group interventions among patients with Cluster B personality disorder, trauma exposure, or difficulties in emotion regulation [54, 59, 60]. Therefore, alongside outcome evaluation, the present study aimed to examine baseline psychological and sociodemographic predictors of dropout from the MBCT-NSSI programme.

The following hypotheses were formulated:

#### H1

Participation in the MBCT-NSSI intervention will be associated with significant improvements in mindfulness, self-compassion, and adaptive emotion regulation strategies, and reductions in NSSI frequency, impulsivity, depressive symptoms, dissociation, and difficulties in emotion regulation.

#### H2

Improvements in outcomes assessed at all three time points (T1–T3) will be greater during the intervention period (T2–T3) than during the waiting-list period (T1–T2).

#### H3

Treatment dropout will be more likely among participants with greater baseline vulnerability (e.g., higher depression, dissociation, and impulsivity; lower emotion regulation capacity and self-compassion), as well as younger age.

## Methods

### Participants and procedure

Adult psychiatric outpatients diagnosed with BPD and reporting NSSI within the past six months were recruited between January 2019 and June 2023 from local outpatient clinics. Exclusion criteria included acute suicidal crisis, current psychosis, manic episode, severe substance use disorder, organic brain disorder, or intellectual disability. Eligibility was assessed through clinical interviews and medical documentation. Diagnostic confirmation was based on the BPD module of the Structured Clinical Interview for DSM-IV Axis II Disorders [61, 62], conducted by trained clinical psychologists under supervision. All participants provided written informed consent prior to participation, per the Declaration of Helsinki, and the study received institutional ethical approval.

Prior to enrolment, candidates completed a brief semi-structured screening interview based on the MBCT suicide prevention protocol by Williams et al. [39], adapted to focus on NSSI rather than suicidal ideation. The interview served (i) an orientation/screening purpose by assessing motivation and practical feasibility (e.g., ability to commit to session attendance and home practice), and (ii) a clinical assessment purpose by evaluating NSSI severity and key characteristics. Additional contextual information (e.g., situational antecedents and perceived functions of NSSI) was collected to inform clinical understanding and group fit, but these data were not analysed quantitatively in the present manuscript.

The target sample size was determined based on methodological guidance for pilot studies. Viechtbauer et al. [63] recommend that a minimum of 45 participants would be sufficient to detect medium effects with 90% confidence. Importantly, our power calculation was based not on the number of participants enrolled at baseline, but on the number who completed the intervention and the post-assessment (T3). Intervention groups were conducted consecutively, and data collection continued until the minimum number of 45 completers at T3 was reached. Recruitment was closed once this threshold was met, resulting in a final T3 sample of exactly 50 participants. Accordingly, all participants who completed baseline assessment (T1) before the recruitment closure were included in the analysis.

Of the 158 individuals screened, 120 met the inclusion criteria and completed baseline assessments. A total of 50 participants completed the full intervention and post-assessments, constituting the final sample. Dropout was defined as non-completion of the post-intervention assessment (T3), resulting either from discontinuation due to exceeding the absence limit in line with the programme participation agreement (> 2 absences; attending < 7/9 sessions) or from failure to complete T3 despite adequate attendance.

The average age in the eligible sample (*N* = 120) was 27,68 years (SD = 7.78); the sample included 111 women (92.5%) and nine men (7.5%). Most participants (75.83%, *N* = 91) had at least one comorbid diagnosis, and 23.17% had two or more (see Table 1). More than half (57.1%) were receiving psychiatric medication at the time of the study. Previous suicide attempts were reported by 44.2%, and 23.8% reported suicide in a first-degree relative.

**Table 1:** Prevalence of comorbid disorders in the BPD sample

Comorbid diagnosis (ICD-10)		%	∑%
Depression	Bipolar (F31.3, F31.6, F31.8)	13.33	43.33
	Unipolar (F32.0, F32.1, F32.2, F32.8)	20.00	
	Recurrent/persistent (F33.0, F33.1, F34.8)	10.00	
Neurotic, stress-related, and somatoform disorders	Mixed anxiety and depressive disorder (F41.2)	20.00	40.83
	Anxiety disorders (F40.0-F41.8, without F41.2)	12.50	
	Obsessive-compulsive disorder (F42.0-F42.2)	2.50	
	Post-traumatic stress disorder (F43.1)	5.83	
Eating disorders (F50.0, F50.2, F50.8)		11.67	11.67
Comorbid personality disorder		18.33	18.33
Other (F19.1 in remission)		4.17	4.17

#### Treatment as usual and concurrent care 

Participants received treatment as usual (TAU) during both the waiting period and the active MBCT-NSSI phase. TAU was not stratified or recorded in detail (e.g., individual psychotherapy vs. psychiatric follow-up and medication management only), and we therefore did not differentiate outcomes by type or intensity of concurrent care. Concurrent group psychotherapy was not permitted, and no participant attended parallel group treatment during either the waiting-list or intervention phase. Participants were asked to report any major changes in treatment; no participant reported initiating a new individual psychotherapy between enrolment (T1) and post-treatment (T3). Psychiatric hospitalisation automatically resulted in discontinuation from the study (see participant flow diagram), regardless of whether it occurred during the waiting (*n* = 6) or intervention (*n* = 2) phase.

### Study design

The study employed a nonrandomised pre–post design with a repeated-measures waitlist control period, which served only as a *within-subject comparator*, not an independent control group. Reporting followed the Transparent Reporting of Evaluations with Nonrandomized Designs (TREND) 2024 checklist to ensure methodological transparency and reproducibility.

**Fig. 1:**
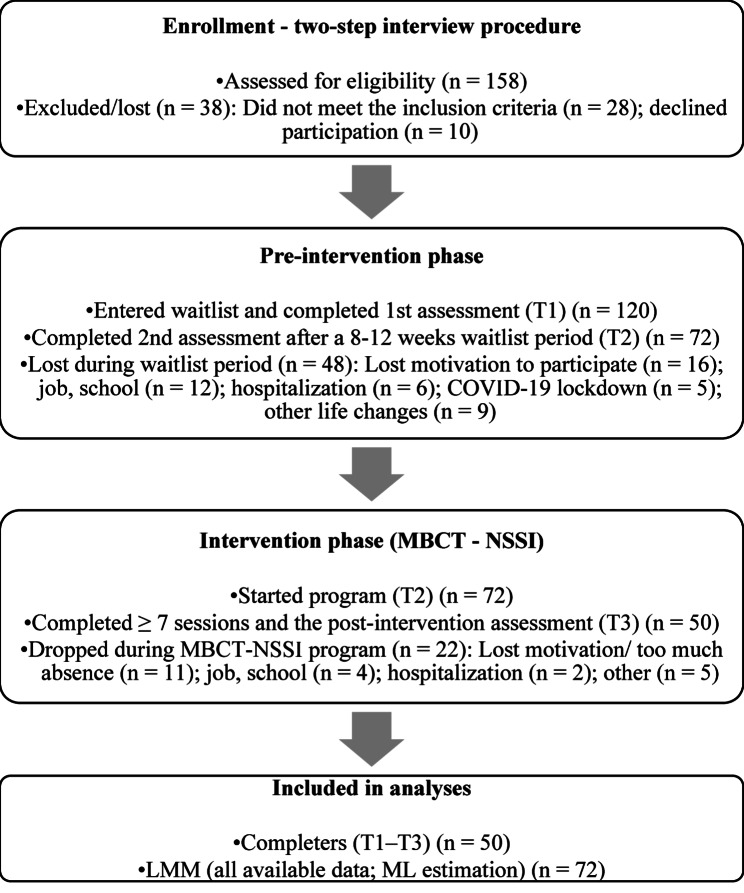
Participant flow through the study based on CONSORT-adapted guidelines for nonrandomised trials

Assessments were conducted at three time points: baseline, 8–12 weeks before the intervention (T1); pre-intervention (T2); and post-intervention (T3). Only a small number of participants (*n* = 7) completed a follow-up at 6 months, which was insufficient for statistical analysis and was thus excluded. The design did not include a control group. Participant recruitment, inclusion, and attrition are illustrated in Fig. 1, which follows CONSORT-adapted guidelines for nonrandomised trials.

### Intervention

The participants attended a 9-week MBCT-NSSI group intervention, adapted from the MBCT suicide prevention protocol by Williams et al. [39]. Each 90-minute session was co-facilitated by clinical psychologists trained in MBCT. The first two authors and the last author served as group leaders; the last author also supervised all intervention groups to ensure treatment fidelity and adherence to the MBCT protocol. To ensure protocol fidelity, all sessions followed pre-defined plans specifying the formal and informal mindfulness practices for each meeting. Guided meditations were delivered verbatim from pre-written scripts. Therapists recorded session notes to track adherence, which were reviewed during weekly supervision. Across six MBCT-NSSI groups, only three sessions required rescheduling of a practice. Despite these minor deviations, the full protocol content was delivered. The MBCT-NSSI protocol is the intellectual property of the Department of Clinical Psychology, Semmelweis University.

The programme incorporated psychoeducation, formal and informal mindfulness practices, structured homework assignments, and action plans. Two sessions included loving-kindness meditation, based on its potential benefits for self-compassion and emotion regulation. The participants received a workbook containing educational materials, meditation logs, and weekly assignments. Weekly reminder emails provided audio/video guidance for home practice. Session content included mindfulness of thoughts/emotions, experiential avoidance, self-compassion, and relapse prevention. The final session focused on consolidating skills and future planning [29]. The MBCT-NSSI programme was based on standard MBCT principles and adapted for individuals with borderline personality disorder and ongoing non-suicidal self-injury; key adaptations are described in the Discussion.

### Measures

Assessments included (1) a demographic and clinical questionnaire (completed at T1 only); (2) a semi-structured NSSI interview (T1 and T3); and (3) a battery of validated self-report scales assessing psychological mechanisms (T1, T2, T3).

NSSI was assessed using structured clinical interview questions that covered types and frequency over the past 6 months at baseline (T1) and over the 9-week intervention period at post-assessment (T3). These differing reference periods limit the direct comparability of scores across time points, particularly regarding frequency estimates. Frequency responses were categorised as follows: [1] *several times a year* [2], *approximately monthly (more than 10 times a year)* [3], *approximately weekly*, and [4] *daily or more frequent*. To allow for more accurate detection of change, a fifth category— *(0) no self-injury since the start of the group*—was created for post-intervention analysis (T3). Because all participants met the inclusion criteria requiring recent NSSI, no participant reported no self-injury in the past six months at baseline (T1). This modification ensured that participants with low-frequency baseline NSSI who completely ceased the behaviour after the intervention were accurately captured in the analyses.

The interview guide was adapted from the MBCT suicide prevention protocol described by Williams et al. (2015) and operationalised for NSSI by the study team. A standardised prompt list and coding rules were used across groups and assessment points; the extract of the interview guide (English translation) is provided in Supplementary Material S1 to support replicability. The interview was administered by trained clinical psychologists; no formal interrater reliability or audio-recorded fidelity checks were conducted.

The following self-administered questionnaires were included in the statistical data analysis:

- **General datasheet**: questions related to demographic information, psychiatric history, number and method of former suicide attempts (only at T1).
- **Rosenberg Self-Esteem Scale (RSES-H)**: is a 10-item scale that measures global self-worth on a 4-point Likert scale ranging from 0 (strongly disagree) to 3 (strongly agree). We used the scale as unidimensional [64, 65].
- **Five-Facet Mindfulness Questionnaire (FFMQ)**: A 39-item self-report questionnaire used to measure mindfulness level as a personality trait [50]. The five subscales of the questionnaire are: *observing*,* describing*,* acting with awareness*,* nonjudging of inner experience*, and *nonreactivity to inner experience*. The adapted Hungarian [66] version is under the standardisation process.
- **Beck Depression Inventory – Shortened version (BDI-S)**: a 9-item self-measure scale that measures the severity of depression [67, 68].
- **Beck Hopelessness Scale – Shortened version (BHS-S)**: 4-item shortened scale of the original Beck Hopelessness Scale for measuring hopelessness [69, 70].
- **Barratt Impulsivity Scale-Shortened (BIS-8)**: an 8-item self-report questionnaire to measure impulsivity on a four-point Likert scale [71, 72].
- **Dissociative Experiences Scale (DES)**: a 28-item self-report questionnaire measuring the frequency of dissociative experiences on a scale of 0-100 [73, 74].
- **Cognitive Emotion-Regulation Questionnaire (CERQ)**: A 36-item scale evaluating nine cognitive strategies for emotion regulation. Subscales are categorised as adaptive (e.g., *putting into perspective*,* positive refocusing*, and *acceptance*) and maladaptive (e.g., s*elf-blame*,* rumination*) [75–77].
- **Self-compassion Scale (SCS)**: A 26-item measure assessing three bipolar components: *self-judgement* vs. *self-kindness*,* isolation* vs. *common humanity*, and *over-identification* vs. *mindfulness* [78, 79].
- **Structured Clinical Interview for DSM-IV Axis II Disorders**,** Borderline Personality Disorder subscale (SCID-II-BPD)**: used to confirm BPD diagnosis and assess symptom severity [61, 62].

### Statistical analysis

All analyses were performed using IBM SPSS Statistics, version 28. Statistical significance was defined as *p* < 0.05 (two-tailed). Bonferroni correction was applied to pairwise comparisons and to the dropout logistic regression models to control for multiple testing. Primary outcomes were NSSI frequency and trait mindfulness (FFMQ). Secondary outcomes were self-compassion (SCS), self-esteem (RSES), impulsivity (BIS-8), depression (BDI-S), dissociation (DES), and cognitive emotion regulation (CERQ).

Ordinal outcomes (e.g., NSSI frequency) were analysed using the Wilcoxon signed-rank test. Continuous outcomes (e.g., RSES, FFMQ, BDI) were analysed using linear mixed-effects models (LMM) with random intercepts, an AR (1) covariance structure, and time as the fixed factor. Little’s MCAR test suggested a completely random missingness pattern (χ²(139) = 76.01, *p* = 1.00); however, this test has low power in small longitudinal clinical samples. LMM were estimated by maximum-likelihood (ML), which is appropriate under the missing-at-random (MAR) assumption. The exact duration of the waitlist period was not systematically recorded for each participant; therefore, analyses could not statistically control variability in waiting time (8–12 weeks).

Several baseline variables (e.g., impulsivity, maladaptive emotion regulation, and self-compassion) were significantly associated with dropout, suggesting that missingness was more consistent with MAR than MCAR. These predictors were not included in the outcome models, which weakens support for the MAR assumption. No sensitivity analyses (e.g., multiple imputation or pattern-mixture models) were conducted, further limiting robustness.

To identify baseline predictors of dropout, binary logistic regression models were fitted with completion status as the dependent variable. Dropout analyses were exploratory. Psychological predictors were analysed separately from sociodemographic variables to reduce model complexity and potential overfitting. Multicollinearity was assessed via Pearson’s correlations and variance-inflation factors (VIFs). Because hopelessness was highly correlated with depression (*r* = 0.68), it was excluded from multivariate models. All remaining predictors showed acceptable collinearity (*r* < 0.51, VIF = 1.03–1.77).

## Results

### Baseline characteristics (T1)

All scales demonstrated acceptable internal consistency (Cronbach’s α > 0.70), consistent with guidelines for early-stage studies [80]. Participants reported elevated depressive symptoms, hopelessness, impulsivity, and dissociation, along with reduced mindfulness, self-esteem, and self-compassion (Table 2).

**Table 2:** Healthy standard values/cut-off points and descriptive statistics for each psychometric instrument at the baseline assessment (T1)

Measures (*N* = 120)	Mean (SE)		Range		Reference Values (Normative Means / Clinical Cut-offs)
RSES	10.49 (0.51)	0–30		> 15 (65)	
FFMQ	105.32 (1.48)	39 − 195		*M* = 133.80, *SD* = 21.58 (72)	
CERQ_ad	51.72 (1.25)	20–100		*M* = 64.57, *SD* = 10.33 (77)	
CERQ_mad	50.53 (1.02)	16–90		*M* = 39.04, *SD* = 8.01 (77)	
BDI-S	21.78 (0.48)	9–36		< 19 (67)	
BHS-S	10.25 (0.34)	4–16		< 9 (70)	
BIS-8-S	20.65 (0.39)	8–32		*M* = 15.46, *SD* = 4.98 (72)	
DES	766.63 (42.95)	0–2800		-	
SCS	53.36 (1.42)	26–130		*M* = 70.31, *SD* = 12.11 (79)	

Notes: Reference values (normative means/clinical cut-offs) are taken from the cited validation/standardisation studies (see references in table) and are provided for descriptive context only. They were not used for inferential comparison with the present BPD sample. RSES = Rosenberg Self-Esteem Scale, FFMQ = Five-Facet Mindfulness Questionnaire, CERQ = Cognitive Emotion Regulation Questionnaire, CERQ_ad = CERQ adaptive strategies subscale, CERQ_mad = CERQ maladaptive strategies subscale, BDI-S = Beck Depression Inventory Shortened, BHS-S = Beck Hopelessness Inventory Shortened, BIS-8-S = Barratt Impulsivity Scale Shortened, DES = Dissociative Experience Scale, SCS=Self-Compassion Scale

Before the intervention, 84% of the participants engaged in NSSI at least monthly, with 18% reporting daily and 22% weekly NSSI. 56% of the participants engaged in multi-method NSSI, and 19% in three or more methods. Skin-cutting (54%) and self-hitting (44%) were the most common methods.

### Changes in outcomes over time

#### Non-suicidal self-injury (NSSI)

NSSI frequency rates differed significantly between baseline (T1) and post-intervention (T3) assessments (Wilcoxon’s Z [49]=-5.639, *p* < 0.001, N_decrease_=36, N_unchanged_=14). Post-intervention, only 2% of participants reported daily NSSI, 6% weekly, 38% monthly, and 54% less frequently than monthly. Notably, eight participants who engaged in low-frequency self-harm at baseline reported no NSSI at post-intervention assessment. Given the absence of intermediate (T2) and follow-up NSSI assessments, conclusions regarding intervention specificity or durability cannot be drawn.

#### Mindfulness and secondary outcomes

LMM were conducted to assess change across three time points: baseline (T1), pre-intervention (T2), and post-intervention (T3). The estimated marginal means (95% CIs), F-Tests, and pairwise comparisons from LMM are presented in Table 3. A Bonferroni correction for nine comparisons set the significance at *p* < 0.0056.

Mindfulness (FFMQ) as primary outcome and self-compassion (SCS) as secondary outcome increased significantly following MBCT-NSSI (both *p* < 0.001), each reflecting large post-intervention effects (d = 0.84 and 0.80), with no change during the waiting period (*p* = 0.954, *p* = 0.754). Self-esteem (RSES) also improved significantly from T2 to T3 (*p* < 0.001, d = 0.67), whereas no significant change occurred during the waiting period after Bonferroni correction (*p* = 0.039 > 0.0056).

Adaptive emotion-regulation strategies (CERQ-ad) remained stable across assessments. In contrast, maladaptive strategies (CERQ-mad) did not show a significant change during the waiting period (*p* = 0.263), followed by a significant post-intervention decrease (*p* = 0.002, d = 0.47).

Further secondary outcomes also demonstrated significant time effects (see Table 3). Depressive symptoms (BDI-S) declined across assessments, with no change during the waiting period (*p* = 0.245) but a moderate reduction from T2 to T3 (d = 0.50, *p* < 0.001). Hopelessness (BHS-S) similarly decreased following MBCT-NSSI (*p* = 0.001, d = 0.46).

Impulsivity (BIS-8-S) exhibited significant changes over time (*p* < 0.001), with an increase observed during the waiting period (d = 0.56), followed by a notable decrease following the intervention (d = 0.69), resulting in a net reduction at post-treatment.

Dissociative symptoms (DES) also declined over time (*p* = 0.006). Although the T2–T3 difference did not meet the Bonferroni-adjusted significance threshold (*p* = 0.047 > 0.0056), the downward pattern across assessments suggests a numerical improvement.

**Table 3:** Linear mixed models (LMM) results for mindfulness skills and secondary outcomes

Measures	Mean (95% CI)			F(df)	*p*	Bonferroni pairwise comparison			
	T1 (*N* = 120)	T2 (*N* = 72)	T3 (*N* = 50)			*p* (T1-T2)	d	*p* (T2-T3)	d
FFMQ	105.32 (102.39–108.24)	106.69 (103.35–110.03)	120.17 (116.30–124.05)	38.82 (2, 100.5)	**< 0.001**	0.954	—	**< 0.001**	0.84
RSES	10.49 (9.49–11.49)	11.55 (10.43–12.67)	13.62 (12.35–14.89)	15.56 (2, 93.7)	**< 0.001**	0.039	—	**< 0.001**	0.67
CERQ_ad	51.72 (49.26–54.18)	50.72 (47.93–53.51)	53.19 (49.92–56.45)	1.94 (2, 95.1)	0.150	1.000	—	0.194	—
CERQ_mad	50.53 (48.51–52.56)	51.91 (49.67–54.15)	48.61 (46.04–51.19)	6.80 (2, 97.9)	**0.002**	0.263	—	**0.002**	0.47
BDI-S	21.78 (20.84–22.73)	21.11 (20.06–22.16)	19.06 (17.86–20.25)	13.69 (2, 90.0)	**< 0.001**	0.245	—	**< 0.001**	0.50
BHS-S	10.25 (9.59–10.91)	10.13 (9.41–10.84)	9.12 (8.32–9.92)	7.82 (2, 93.5)	**0.001**	1.000	—	**0.001**	0.46
BIS-8-S	20.65 (19.87–21.43)	21.70 (20.86–22.54)	19.69 (18.77–20.62)	22.18 (2, 90.0)	**< 0.001**	**< 0.001**	0.56	**< 0.001**	0.69
DES	766.63 (681.66–851.59)	717.79 (627.65–807.92)	644.94 (545.99–743.89)	5.48 (2, 91.1)	0.006	0.154	—	0.047	—
SCS	53.36 (50.56–56.16)	55.04 (51.77–58.31)	63.79 (59.92–67.67)	15.14 (2, 132.3)	**< 0.001**	0.754	—	**< 0.001**	0.80

Notes: RSES = Rosenberg Self-Esteem Scale, FFMQ = Five-Facet Mindfulness Questionnaire, CERQ = Cognitive Emotion Regulation Questionnaire, CERQ_ad = CERQ adaptive strategies subscale, CERQ_mad = CERQ maladaptive strategies subscale, BDI-S = Beck Depression Inventory Shortened, BHS-S = Beck Hopelessness Inventory Shortened, BIS-8-S = Barratt Impulsivity Scale Shortened, DES = Dissociative Experience Scale, SCS = Self-Compassion Scale. d = Cohen’s d effect size for pairwise comparisons (T1–T2, T2–T3)

Missing effect sizes (—) indicate non-significant comparisons, or d was not computed due to lack of significance

Bonferroni correction for multiple tests was used; p < 0.0056 was considered significant

### Dropout predictors

To reduce model complexity and the risk of overfitting, baseline predictors of dropout were analysed in two separate binary logistic regression models using the Wald backward stepwise method: one including eight psychological predictors (mindfulness, self-compassion, depression, hopelessness, impulsivity, adaptive and maladaptive emotion regulation, dissociation), and another including five sociodemographic/clinical predictors (age, sex, number of comorbid diagnoses, history of suicide attempts, and suicide in the close family). Given the number of predictors, a Bonferroni correction was applied within each model, resulting in adjusted significance thresholds of *p* < 0.00625 and *p* < 0.01, respectively.

In the psychological model, the overall fit was acceptable (Hosmer–Lemeshow χ² [8] = 8.25, *p* = 0.409; Nagelkerke R² = 0.305). Using the Wald backward stepwise method, after Bonferroni correction, three variables remained significant predictors of dropout (Table 4): higher impulsivity (BIS-8-S; OR = 1.20, 95% CI = 1.08–1.35), greater use of maladaptive cognitive emotion regulation strategies (CERQ_mad; OR = 1.08, 95% CI = 1.03–1.12), and, unexpectedly, higher self-compassion (SCS; OR = 1.06, 1.02–1.11). Adaptive cognitive emotion regulation strategies (CERQ_ad) showed a trend (*p* = 0.008) but did not meet the corrected threshold.

In the sociodemographic model, the model fit was acceptable (Hosmer–Lemeshow χ² [7] = 11.26, *p* = 0.128; Nagelkerke R² = 0.224). Only one variable, *fewer comorbid diagnoses*, significantly predicted dropout (OR = 0.40, 95% CI = 0.23–0.69) after correction (Table 4). Younger age (*p* = 0.039) and history of suicide attempts (*p* = 0.061) showed non-significant trends.

Due to the number of predictors tested and the small sample size, results should be interpreted with caution.

**Table 4:** Binary logistic regression predicting intervention dropout

Predictor	B	SE	Wald	OR (Exp(B))	95% CI for OR	*p*
**Psychological predictors model**						
CERQ_ad	-0.051	0.019	7.02	0.95	0.92–0.99	**0.008**
CERQ_mad	0.073	0.024	9.56	1.08	1.03–1.12	**0.002**
BIS-8-S	0.186	0.056	12.34	1.20	1.08–1.35	**0.001**
SCS	0.059	0.021	8.02	1.06	1.02–1.11	**0.005**
**Sociodemographic and clinical characteristics predictors model**						
Age	-0.056	0.027	4.28	0.95	0.90–1.00	0.039
Number of comorbid diagnoses	-0.929	0.286	10.56	0.40	0.23–0.69	**< 0.001**
Former suicide attempts	0.823	0.440	3.52	2.28	0.96–5.38	0.061

Notes: Psychological predictor model fit: Cox & Snell R² = 0.227, Nagelkerke R² = 0.305; Hosmer–Lemeshow χ² [8] = 8.25, *p* = 0.409. Bonferroni correction for multiple tests was used; *p* < 0.0063 can be considered significant

Sociodemographic and clinical characteristics predictors model: Cox & Snell R² = 0.168, Nagelkerke R² = 0.224; Hosmer–Lemeshow χ²(7) = 11.26, p = 0.128. Bonferroni correction for multiple tests was used; p < 0.01 can be considered significant

CERQ = Cognitive Emotion Regulation Questionnaire, CERQ_ad = CERQ adaptive strategies subscale, CERQ_mad = CERQ maladaptive strategies subscale, BIS-8-S = Barratt Impulsivity Scale Shortened, SCS=Self-Compassion Scale

## Discussion

This within-subject pre–post pilot study evaluated the feasibility and preliminary outcomes of a mindfulness-based cognitive therapy intervention tailored for individuals with borderline personality disorder (MBCT-NSSI). The core characteristics of BPD include being overwhelmed and carried away by emotions and impulses, and the pervasive lability and shame that accompany them. Mindfulness-based approaches address these vulnerabilities by enhancing present-moment awareness, non-judgmental acceptance, and self-regulation [36, 37]. Therefore, MBCT might be especially appropriate for BPD. To our knowledge, this is the first study to examine a structured, adapted MBCT programme targeting NSSI in individuals with BPD using a within-subject design with a waiting-period comparator. We examined whether participation in MBCT-NSSI was associated with changes in self-injury and core psychological mechanisms such as mindfulness, emotion regulation, and impulsivity (H1); whether changes in outcomes were larger during the intervention period compared to the waiting-period phase for outcomes assessed at all three time points (H2); and whether baseline psychological or clinical factors predicted treatment dropout (H3).

### Hypothesis 1: Improvements following MBCT-NSSI

In line with our first hypothesis, participation in the MBCT-NSSI intervention was associated with significant improvements in several key clinical and psychological domains. Most notably, participants reported a marked reduction in NSSI frequency between baseline and post-intervention assessment, with the majority decreasing the frequency of self-harm to monthly or less frequent occurrences (Wilcoxon’s Z [49]=-5.639, *p* < 0.001, N_decrease_=36, N_unchanged_=14). Considering that NSSI is a highly resistant and high-risk symptom of BPD, even a moderate reduction represents a clinically meaningful change. Although the structured NSSI interview was administered only at T1 and T3, conclusions regarding the specificity of interventions for NSSI outcomes should be interpreted with caution.

Beyond behavioural outcomes, significant improvements were observed in several targeted psychological capacities between T2 (pre-intervention) and T3 (post-intervention), following the active treatment phase. Participants demonstrated statistically and clinically significant increases in mindfulness (d = 0.84) and self-compassion (d = 0.80), with a large effect size, alongside a moderate, clinically meaningful improvement in self-esteem (d = 0.67) (*p* < 0.001 in all cases). In parallel, depressive symptoms, hopelessness, and impulsivity showed moderate reductions (ds ≈ 0.46–0.69, *p* ≤ 0.001 in all cases; Table 3), indicating clinically relevant change. For outcomes assessed at all three time points, these effects were largely absent during the waiting period (T1–T2), suggesting that improvements were primarily observed during the intervention phase.

The observed increase in psychological protective factors (e.g., mindfulness, self-compassion, self-esteem) aligns with the broader literature on mindfulness-based interventions, which have been shown to promote emotion regulation, reduce reactivity, and enhance self-related processes [40, 42, 44–47, 50].

However, the findings related to emotion regulation strategies were more nuanced. While maladaptive cognitive emotion regulation strategies (CERQ_mad) decreased moderately from pre- to post-intervention (d = 0.47, *p* = 0.002), adaptive strategies (CERQ_ad) did not show improvement. This asymmetry may reflect the nature of MBCT, which emphasises the cultivation of meta-awareness and non-judgmental acceptance rather than teaching explicit cognitive reappraisal techniques. It is possible that reductions in maladaptive responses occurred through enhanced awareness and decentering, without a corresponding increase in overt use of adaptive strategies. Alternatively, the lack of significant change in adaptive strategies may reflect limitations of the CERQ measure in capturing the more experiential and non-cognitive regulatory shifts promoted by MBIs.

Dissociation showed a downward trend across the three assessment points, with significant overall time effects but only trend-level changes from pre- to post-intervention after Bonferroni correction. This pattern should be interpreted cautiously but is encouraging, given that dissociation has been described as one of the most challenging symptoms in BPD and is often associated with poor treatment response [60].

Informal participant comments during sessions and supervision discussions suggested that grounding exercises and heightened body awareness were perceived as particularly helpful in reducing dissociative episodes (these observations were not collected via a formal qualitative methodology). Participants also frequently reported that establishing a consistent mindfulness practice tended to occur only midway through the programme, and maintaining regular practice after the intervention appeared challenging. Due to the robust nature of dissociation, it is plausible that earlier or more intensive home practices may be necessary to achieve more robust effects. These findings support the recommendation to reinforce at-home practice from the outset of the intervention.

Overall, the observed reductions in clinical symptoms and maladaptive emotion regulation strategies, along with increases in self-compassion and self-esteem, are particularly relevant given the well-established protective role of these factors against self-injury. These findings suggest that MBCT-NSSI may target core maintenance mechanisms of NSSI in individuals with BPD. While not all hypothesised changes reached statistical significance, the pattern of results indicates meaningful therapeutic gains in a clinically complex and high-risk population.

### Hypothesis 2: Control condition changes

Our second hypothesis (H2) was supported, as improvements across outcomes were substantially greater during the intervention phase (T2–T3) than during the waiting-list period (T1–T2). During the waiting period, minimal or no positive changes were observed across most outcomes (*p* > 0.0056 in nearly all cases). The only significant change observed during this phase was an increase in impulsivity; however, this reflected a deterioration rather than an improvement (d = 0.56, *p* < 0.001). In contrast, participants demonstrated significant and clinically meaningful improvements across multiple domains following the MBCT-NSSI intervention. Specifically, reductions were observed in depression and impulsivity, alongside increases in mindfulness, self-esteem, and self-compassion. This pattern of results suggests that the observed changes in outcomes assessed at all three time points are unlikely to be explained by time effects, regression to the mean, or repeated measurement, and are consistent with changes occurring during the intervention phase. A reduction in NSSI frequency was also observed between baseline and post-intervention assessment; however, due to the absence of waiting-period NSSI data, phase-specific effects for NSSI cannot be determined.

### Hypothesis 3: Dropout predictors

To examine predictors of dropout, two logistic regression models were tested separately for psychological and sociodemographic variables, with Bonferroni correction applied to control for multiple comparisons.

In the psychological model, higher impulsivity, greater use of maladaptive emotion regulation strategies, and—unexpectedly—higher self-compassion significantly predicted dropout. The associations with impulsivity and maladaptive regulation are consistent with prior findings suggesting that individuals with greater emotional dysregulation may struggle to engage in structured, especially group-based, interventions [54]. The association with self-compassion, although statistically significant, runs counter to theoretical expectations and should be interpreted with caution. It may reflect suppression effects, distorted self-reporting, or state-related fluctuations in self-concept, which are common in BPD populations [81, 82].

In the sociodemographic model, fewer comorbid diagnoses predicted dropout. This result may reflect that individuals with more complex clinical histories are more treatment-engaged due to a higher need or prior exposure to care. Other variables, such as younger age and suicidal history, showed trend-level effects but did not reach significance after correction.

While the dropout rate of the active phase (28.6%) aligns with previous findings in BPD samples [37, 48, 54, 55], the overall rate was much higher, 58,3%. Furthermore, both models showed modest explanatory power (Nagelkerke R² = 0.305 and 0.224, respectively). Suggesting that additional unmeasured factors—such as therapeutic alliance, the outpatient setting’s structure, trauma history, educational level, or overall functioning—may play a more substantial role in treatment retention [53, 54, 57].

In summary, these findings provide initial insights into potential predictors of dropout in MBCT for BPD. However, they should be interpreted cautiously due to the limited sample size, high dropout rate, and the risk of Type I error despite adjustments for multiple comparisons.

### Clinical implications

Participants presented with a clinically severe baseline profile, including frequent and multi-method non-suicidal self-injury, underscoring the need for accessible and targeted interventions for this high-risk population. Accordingly, the findings of this pilot study suggest that a brief, group-based MBCT programme adapted for individuals with BPD (MBCT-NSSI) may represent a feasible and potentially beneficial adjunctive intervention. Improvements were observed across several self-reported outcomes during the intervention period, including reductions in NSSI frequency, depressive symptoms, impulsivity, and hopelessness, alongside increases in mindfulness skills, self-esteem, and self-compassion. For self-reported outcomes assessed at all three time points, these changes were largely absent during the waiting period, supporting the clinical relevance of the active treatment phase.

However, due to the absence of structured NSSI interview data at the pre-intervention time point (T2), the specificity of MBCT-NSSI effects on self-injurious behaviour cannot be conclusively established. Accordingly, these findings should be interpreted as preliminary and hypothesis-generating, emphasising feasibility and short-term clinical signal rather than definitive efficacy. While changes in dissociation and adaptive cognitive emotion regulation strategies were limited, the intervention appeared to primarily reduce maladaptive processes such as impulsivity, self-criticism, and catastrophic thinking—mechanisms often implicated in maintaining NSSI. These effects align with the theoretical goals of MBCT, which aim to increase metacognitive awareness and acceptance.

Given the short duration of the intervention (nine sessions), these changes are clinically meaningful, particularly considering the complexity and high-risk profile of the sample. In contexts with limited access to long-term therapy, MBCT-NSSI may serve as a feasible, lower-intensity treatment option or preparatory intervention, potentially improving psychological readiness for more intensive care.

However, the high overall dropout rate, especially before program initiation, underscores the need for careful pre-treatment screening, enhanced motivational strategies, and perhaps additional engagement supports—particularly for younger patients and those with elevated impulsivity or emotion regulation difficulties. Although dropout during active treatment was comparable to other BPD interventions, future implementations should prioritise strategies to improve retention and continuity, especially in outpatient settings.

Although the present intervention was grounded in standard MBCT principles, MBCT-NSSI differed from traditional MBCT in several clinically relevant ways [29]. The programme was explicitly adapted for individuals with BPD and ongoing NSSI, with a stronger emphasis on safety, affect tolerance, and present-moment awareness of self-harm urges. Session content was simplified, experiential exercises were shorter and more structured, and group processes were more actively contained to reduce emotional overwhelm. Unlike standard MBCT, which typically targets relapse prevention in depression, MBCT-NSSI was designed as a brief adjunctive intervention focusing on self-harm reduction and engagement rather than symptom remission.

The intervention followed a structured session framework developed by the authors. Although a formally published manual is not yet available, the corresponding author can provide session outlines and core worksheets upon reasonable request.

Finally, while these findings support the clinical utility of MBCT-NSSI, the absence of a randomised controlled design, follow-up data, and an active control group limits the strength of the conclusions. Replication in larger, more diverse samples—with extended follow-up—is needed to determine the effects’ sustainability, generalisability, and how MBCT-NSSI compares to other brief interventions for self-harming individuals with BPD.

### Limitations

Several limitations should be considered when interpreting the findings. The study employed a within-subject pre–post design without an active control group, which limits causal inference and increases susceptibility to confounding variables. Due to logistical constraints, the structured NSSI interview was administered only at T1 and T3, preventing a formal statistical comparison of NSSI changes during the waiting period (T1–T2). Furthermore, the NSSI assessment periods differed across time points (6 months at T1 vs. 9 weeks at T3), which complicates the interpretation of observed changes in frequency. This asymmetry may have affected the estimation of effect size and the detection of change. As such, conclusions regarding the specificity of interventions for NSSI outcomes should be interpreted with caution.

The reliance on self-report measures may have introduced bias, particularly in a population characterised by instability in self-concept, which can affect the reliability of responses. Although the assessment time points were structured, the lack of a follow-up assessment precludes conclusions about the durability of the effects. Moreover, the exact duration of the waitlist period was not systematically recorded and therefore could not be statistically controlled.

The high attrition rate also raises concerns about the validity of longitudinal results. Although LMM can handle incomplete data under the MAR assumption, several baseline variables predicted dropout but were not included in the outcome models. Furthermore, no sensitivity analyses were conducted to examine the robustness of the findings under different missing data mechanisms, which limits confidence in the estimates.

Another limitation of the study is the lack of a blinding procedure. Several authors (the first two and the last) also acted as group facilitators, and the last author served as the supervisor of all the groups. Although this may raise concerns about researcher allegiance, verifying the statistical analyses by independent authors mitigated potential analytic bias.

Additionally, while Bonferroni correction was applied to reduce Type I error, the overall sample size—particularly for the dropout analyses—was modest relative to the number of predictors tested, increasing the risk of Type II error or overfitting. Finally, the study was conducted in a single clinical setting with primarily treatment-seeking individuals, which may limit the generalisability of the results to broader BPD or NSSI populations.

### Conclusion

Taken together, this within-subject pre–post pilot study provides preliminary evidence supporting the feasibility of a brief, adapted MBCT-NSSI intervention for individuals with BPD. Improvements were observed in several psychological outcomes assessed across all three time points, including mindfulness, self-compassion, self-esteem, and key clinical symptoms, with changes occurring primarily during the active treatment phase. A reduction in NSSI frequency was also observed between baseline and post-intervention assessment; however, given the absence of structured NSSI data during the waiting period, this finding should be interpreted as preliminary signal detection rather than evidence of intervention-specific or phase-specific effects.

Although dropout rates were high, several psychological and clinical factors predicted engagement, offering guidance for future intervention tailoring. While a 9-week MBCT-NSSI training is not a substitute for long-term psychotherapy tailored to personality disorders, these findings support the feasibility and potential value of structured, mindfulness-based interventions as supplementary support for this high-risk population. Replication in larger, controlled trials with follow-up assessments will be essential to establish sustained effects and optimise clinical utility.

## Supplementary Information

Below is the link to the electronic supplementary material.

Supplementary Material 1

Supplementary Material 2

## Data Availability

The de-identified dataset and supporting documentation are available via the Open Science Framework (OSF): [https://osf.io/z94ym](https://osf.io/z94ym). The study was retrospectively registered at (10.17605/OSF.IO/ZUR84) on May 16, 2025.

## Abbreviations

BDI-S: Beck Depression Inventory Shortened
BHS-S: Beck Hopelessness Inventory Shortened
BIS-8-S: Barratt Impulsivity Scale Shortened
BPD: Borderline personality disorder
CERQ: Cognitive Emotion Regulation Questionnaire
CERQ_ad: CERQ adaptive strategies subscale
CERQ_mad: CERQ maladaptive strategies subscale
CONSORT: Consolidated Standards of Reporting Trials
DBT: Dialectical Behaviour Therapy
DES: Dissociative Experience Scale
FFMQ: Five-Facet Mindfulness Questionnaire
FFMQ_a: FFMQ acting with awareness subscale
FFMQ_d: FFMQ describing subscale
FFMQ_nj: FFMQ nonjudging subscale
FFMQ_nr: FFMQ nonreactivity subscale
FFMQ_o: FFMQ observing subscale
LMM: Linear mixed-effects models
MAR: Missing-at-random assumption
MBCT: Mindfulness-based cognitive therapy
MBCT-NSSI: The 9-week group-based mindfulness-based cognitive therapy program tailored to reduce NSSI
MBI: Mindfulness-based interventions
ML: Maximum-likelihood
NSSI: Non-suicidal self-injury
RSES: Rosenberg Self-Esteem Scale
SCID-II-BPD: Structured Clinical Interview for DSM‐IV Axis II Disorders, Borderline Personality Disorder subscale
SCS: Self-Compassion Scale
TAU: Treatment as usual
TREND: Transparent Reporting of Evaluations with Nonrandomized Designs
VIF: Variance-inflation factors

## References

[1] APA. Diagnostic and statistical manual of mental disorders: DSM-5. Arlington. VA, US: American Psychiatric Publishing, Inc.; 2013. pp. 947–xliv.

[2] Paris J. Suicidality in Borderline Personality Disorder. Medicina. 2019;55(6):223. 31142033 10.3390/medicina55060223 PMC6632023

[3] Shah R, Zanarini MC. Comorbidity of Borderline Personality Disorder: Current Status and Future Directions. Psychiatr Clin North Am. 2018;41(4):583–93. 30447726 10.1016/j.psc.2018.07.009

[4] Wupperman P, Fickling M, Klemanski DH, Berking M, Whitman JB. Borderline personality features and harmful dysregulated behavior: the mediational effect of mindfulness. J Clin Psychol. 2013;69(9):903–11. 23460412 10.1002/jclp.21969

[5] Ellison WD, Rosenstein LK, Morgan TA, Zimmerman M. Community and clinical epidemiology of borderline personality disorder. Psychiatr Clin North Am. 2018;41:561–73. 30447724 10.1016/j.psc.2018.07.008

[6] ISSS. International society for the study of self-injury - What is self-injury? 2018.

[7] Nock MK, Favazza AR. Nonsuicidal self-injury: definition and classification. 2009.

[8] Ribeiro JD, Franklin JC, Fox KR, Bentley KH, Kleiman EM, Chang BP, et al. Self-injurious thoughts and behaviors as risk factors for future suicide ideation, attempts, and death: a meta-analysis of longitudinal studies. Psychol Med. 2016;46(2):225–36. 26370729 10.1017/S0033291715001804 PMC4774896

[9] Muehlenkamp JJ, Brausch AM. Reconsidering Criterion A for the Diagnosis of Non-Suicidal Self-Injury Disorder. J Psychopathol Behav Assess. 2016;38(4):547–58.

[10] Per M, Schmelefske E, Bouchard S, Khoury B. Unraveling the link between mindfulness facets and self-compassion dimensions with non-suicidal self-injury functions and behaviors. Curr Psychol. 2025. 10.1007/s12144-025-07868-y

[11] Blasco-Fontecilla H, Fernández-Fernández R, Colino L, Fajardo L, Perteguer-Barrio R, de Leon J. The addictive model of self-harming (non-suicidal and suicidal) behavior. Front Psychiatry 2016;7–2016. 10.3389/fpsyt.2016.00008 PMC4734209 26869941

[12] Horváthné Pató I, Kresznerits S, Szekeres T, Zinner-Gérecz Á, Perczel-Forintos D. Investigating suicidal behavior among prisoners in the light of the behavioral addiction approach: results of a multicenter cross-sectional study. Front Psychiatry. 2024;15–2024. 10.3389/fpsyt.2024.1448711 PMC11306188 39119071

[13] Bamelis LLM, Arntz A, Wetzelaer P, Verdoorn R, Evers SMAA. Economic evaluation of schema therapy and clarification-oriented psychotherapy for personality disorders: a multicenter, randomized controlled trial. J Clin Psychiatry. 2015;76(11):e1432–40. 26579561 10.4088/JCP.14m09412

[14] Meuldijk D, McCarthy A, Bourke ME, Grenyer BFS. The value of psychological treatment for borderline personality disorder: Systematic review and cost offset analysis of economic evaluations. PLoS ONE. 2017;12(3):e0171592–e. 28249032 10.1371/journal.pone.0171592 PMC5332029

[15] Richter C, Steinacher B, zum Eschenhoff A, Bermpohl F. Psychotherapy of Borderline Personality Disorder: Can the Supply Meet the Demand? A German Nationwide Survey in DBT Inpatient and Day Clinic Treatment Facilities. Commun Ment Health J. 2016;52(2):212–5. 10.1007/s10597-015-9914-0 26323785

[16] Aviram RB, Brodsky BS, Stanley B. Borderline Personality Disorder, Stigma, and Treatment Implications. Harv Rev Psychiatry. 2006;14:249–56. 16990170 10.1080/10673220600975121

[17] Kealy D, Ogrodniczuk JS. Marginalization of borderline personality disorder. J Psychiatr Pract. 2010;16(3):145–54. 20485102 10.1097/01.pra.0000375710.39713.4d

[18] Knaak S, Mantler E, Szeto A. Mental illness-related stigma in healthcare: Barriers to access and care and evidence-based solutions. Healthc Manage Forum. 2017;30(2):111–6. 28929889 10.1177/0840470416679413 PMC5347358

[19] Oud M, Arntz A, Hermens MLM, Verhoef R, Kendall T. Specialized psychotherapies for adults with borderline personality disorder: A systematic review and meta-analysis. Aust N Z J Psychiatry. 2018;52(10):949–61. 30091375 10.1177/0004867418791257 PMC6151959

[20] Linehan MM. Cognitive-behavioral treatment of borderline personality disorder. New York, NY, US: Guilford Press; 1993. xvii, 558-xvii, p.

[21] Storebø OJ, Stoffers-Winterling JM, Völlm BA, Kongerslev MT, Mattivi JT, Jørgensen MS, et al. Psychological therapies for people with borderline personality disorder. Cochrane Database Syst Rev. 2020(5). 10.1002/14651858.CD012955.pub2 PMC7199382 32368793

[22] Cummings JR, Zhang X, Gandré C, Morsella A, Shields-Zeeman L, Winkelmann J, et al. Challenges facing mental health systems arising from the COVID-19 pandemic: Evidence from 14 European and North American countries. Health Policy. 2023;136:104878. 37611521 10.1016/j.healthpol.2023.104878

[23] Shafran R, Myles-Hooton P, Bennett S, Öst L-G. The concept and definition of low intensity cognitive behaviour therapy. Behav Res Ther. 2021;138:103803. 33540242 10.1016/j.brat.2021.103803

[24] Czégel MJR, Unoka Z, Dudas RB, Demetrovics Z. Rumination in Borderline Personality Disorder: A Meta-analytic Review. J Personal Disord. 2022;36(4):399–412. 10.1521/pedi.2022.36.4.399 35913769

[25] Wolff JC, Thompson E, Thomas SA, Nesi J, Bettis AH, Ransford B, et al. Emotion dysregulation and non-suicidal self-injury: A systematic review and meta-analysis. Eur Psychiatry. 2019;59:25–36. 30986729 10.1016/j.eurpsy.2019.03.004 PMC6538442

[26] Clapham R, Brausch A. Emotion regulation deficits across the spectrum of self-harm. Death Stud. 2022;46(10):2477–84. 34486924 10.1080/07481187.2021.1972366 PMC9554899

[27] Hasking P, Whitlock J, Voon D, Rose A. A cognitive-emotional model of NSSI: using emotion regulation and cognitive processes to explain why people self-injure. Cogn Emot. 2017;31(8):1543–56. 27702245 10.1080/02699931.2016.1241219

[28] Nock MK, Prinstein MJ. A functional approach to the assessment of self-mutilative behavior. J Consult Clin Psychol. 2004;72(5):885–90. 15482046 10.1037/0022-006X.72.5.885

[29] Kresznerits S, Zinner-Gérecz Á, Perczel-Forintos D. [Borderline personality disorder and non-suicidal self-injury: the role of mindfulness training in risk reduction]. Psychiatr Hung. 2023;38(2):142–52. 37439291

[30] Selby EA, Anestis MD, Joiner TE. Understanding the relationship between emotional and behavioral dysregulation: Emotional cascades. Behav Res Ther. 2008;46:593–611. 18353278 10.1016/j.brat.2008.02.002

[31] Per M, Simundic A, Argento A, Khoury B, Heath N. Examining the relationship between mindfulness, self-compassion, and emotion regulation in self-injury. Archives Suicide Res. 2021;1–16. 10.1080/13811118.2021.1885534 33596395

[32] Wupperman P, Neumann CS, Axelrod SR. Do Deficits in Mindfulness Underlie Borderline Personality Features and Core Difficulties? J Personal Disord. 2008;22(5):466–82. 10.1521/pedi.2008.22.5.466 18834295

[33] Wupperman P, Neumann CS, Whitman JB, Axelrod SR. The role of mindfulness in borderline personality disorder features. J Nerv Ment Dis. 2009;197(10):766–71. 19829206 10.1097/NMD.0b013e3181b97343

[34] Peters JR, Eisenlohr-Moul TA, Upton BT, Talavera NA, Folsom JJ, Baer RA. Characteristics of Repetitive Thought Associated with Borderline Personality Features: A Multimodal Investigation of Ruminative Content and Style. J Psychopathol Behav Assess. 2017;39(3):456–66. 28983149 10.1007/s10862-017-9594-x PMC5624311

[35] BogosG, Măirean C, Tiperciuc A, Muntele-Hendreș D, Mariș A-M. The relationship between borderline personality disorder symptoms and mindfulness: a meta-analysis. Mindfulness. 2025;16(11):3087–103 10.1007/s12671-025-02674-w

[36] Duan Y, Lv X. The Application of Mindfulness in The Treatment of Borderline Personality Disorder. Trans Mater Biotechnol Life Sci. 2024;7:241–7.

[37] Schmidt C, Soler J, Vega D, Pascual JC. Practice matters: The role of mindfulness skills in emotion dysregulation in borderline personality disorder. J Context Behav Sci. 2024;32:100756. 10.1186/s40479-024-00265-0 PMC11367780 39218933

[38] Williams M, Teasdale J, Segal Z, Kabat-Zinn J. The mindful way through depression: Freeing yourself from chronic unhappiness. New York, NY, US: Guilford Press; 2007. viii, 273-viii, p.

[39] Williams M, Fennell M, Barnhofer T, Crane R, Silverton S. Mindfulness-based cognitive therapy with people at risk of suicide. Guilford; 2015.

[40] Carmona i Farrés C, Elices M, Soler J, Domínguez-Clavé E, Martín-Blanco A, Pomarol-Clotet E, et al. Effects of mindfulness training on the default mode network in borderline personality disorder. Clin Psychol Psychother. 2019;26(5):562–71. 31132302 10.1002/cpp.2382

[41] Argento A, Simundic A, Mettler J, Mills DJ, Heath NL. Evaluating the Effectiveness of a Brief Mindfulness Activity in University Students With Non-Sicidal Self-Injury Egagement. Archives Suicide Res. 2022;26(2):871–85. 10.1080/13811118.2020.1841052 33135590

[42] Zheng Q, Zhou HY, Li K, Liu Y, Nan W, Gong J. The effectiveness of mindfulness-based intervention for psychological distress and emotion regulation in college students with non-suicidal self-injury. Appl Psychol Health Well Being. 2024;16(4):2083–98. 39032127 10.1111/aphw.12580

[43] Liu P, Li BY, Liu J, Yang XJ, Li Y, Li YY, et al. Implementing twelve-weeks of loving-kindness meditation and mindfulness of breathing for adolescents with nonsuicidal self-injury and their parents: a mixed method pilot study. BMC Psychol. 2025;13(1):18. 40702478 10.1186/s40359-025-03151-8 PMC12288253

[44] Franco C, Amutio A, López-González L, Oriol X, Martínez-Taboada C. Effect of a mindfulness training program on the impulsivity and aggression levels of adolescents with behavioral problems in the classroom. Front Psychol. 2016;7(SEP):1–8. 27713709 10.3389/fpsyg.2016.01385 PMC5031764

[45] Peters JR, Smart LM, Eisenlohr-Moul TA, Geiger PJ, Smith GT, Baer RA. Anger Rumination as a Mediator of the Relationship Between Mindfulness and Aggression: The Utility of a Multidimensional Mindfulness Model. J Clin Psychol. 2015;71(9):871–84. 25919798 10.1002/jclp.22189 PMC12063192

[46] Jankowski T, Holas P. Metacognitive model of mindfulness. Conscious Cogn. 2014;28:64–80. 25038535 10.1016/j.concog.2014.06.005

[47] Calvete E, Royuela-Colomer E, Maruottolo C. Emotion dysregulation and mindfulness in non-suicidal self-injury. Psychiatry Res. 2022;314:114691. 35777277 10.1016/j.psychres.2022.114691

[48] Elices M, Pascual JC, Portella MJ, Feliu-Soler A, Martín-Blanco A, Carmona C, et al. Impact of Mindfulness Training on Borderline Personality Disorder: A Randomized Trial. Mindfulness. 2016;7(3):584–95. 10.1186/s40479-015-0035-8 PMC4709962 26759718

[49] Feliu-Soler A, Pascual JC, Elices M, Martín-Blanco A, Carmona C, Cebolla A, et al. Fostering Self-Compassion and Loving-Kindness in Patients With Borderline Personality Disorder: A Randomized Pilot Study. Clin Psychol Psychother. 2017;24(1):278–86. 26818533 10.1002/cpp.2000

[50] Baer RA, Smith GT, Hopkins J, Krietemeyer J, Toney L. Using self-report assessment methods to explore facets of mindfulness. Assessment. 2006;13(1):27–45. 16443717 10.1177/1073191105283504

[51] Yang Q, Xie R, Li J, Zhang R, Ding W, Li W. The mutual relationship between self-compassion, perceived social support, and adolescent NSSI: a three-wave longitudinal study. Mindfulness. 2023.

[52] Rees CS, Hasking P, Breen LJ, Lipp OV, Mamotte C. Group mindfulness based cognitive therapy vs group support for self-injury among young people: Study protocol for a randomised controlled trial. BMC Psychiatry. 2015;15(1):1–8. 26152135 10.1186/s12888-015-0527-5 PMC4495689

[53] Arntz A, Mensink K, Cox WR, Verhoef REJ, van Emmerik AAP, Rameckers SA, et al. Dropout from psychological treatment for borderline personality disorder: a multilevel survival meta-analysis. Psychol Med. 2023;53(3):668–86. 36453183 10.1017/S0033291722003634 PMC9975988

[54] De Salve F, Rossi C, Gioacchini E, Messina I, Oasi O. Dropout in Psychotherapy for Personality Disorders: A Systematic Review of Predictors. Clin Psychol Psychother. 2025;32(3):e70080. 40325843 10.1002/cpp.70080 PMC12053075

[55] Soler J, Valdepérez A, Feliu-Soler A, Pascual JC, Portella MJ, Martín-Blanco A, et al. Effects of the dialectical behavioral therapy-mindfulness module on attention in patients with borderline personality disorder. Behav Res Ther. 2012;50(2):150–7. 22225697 10.1016/j.brat.2011.12.002

[56] Herzog P, Feldmann M, Voderholzer U, Gärtner T, Armbrust M, Rauh E, et al. Drawing the borderline: Predicting treatment outcomes in patients with borderline personality disorder. Behav Res Ther. 2020;133:103692. 32801095 10.1016/j.brat.2020.103692

[57] Iliakis EA, Ilagan GS, Choi-Kain LW. Dropout rates from psychotherapy trials for borderline personality disorder: A meta-analysis. Personal Disord. 2021;12(3):193–206. 33591777 10.1037/per0000453

[58] Kröger C, Harbeck S, Armbrust M, Kliem S. Effectiveness, response, and dropout of dialectical behavior therapy for borderline personality disorder in an inpatient setting. Behav Res Ther. 2013;51(8):411–6. 23727659 10.1016/j.brat.2013.04.008

[59] Berghuis H, Bandell CC, Krueger RF. Predicting dropout using DSM-5 Section II personality disorders, and DSM-5 Section III personality traits, in a (day)clinical sample of personality disorders. Personal Disord. 2021;12(4):331–8. 32730060 10.1037/per0000429

[60] Arntz A, Stupar-Rutenfrans S, Bloo J, van Dyck R, Spinhoven P. Prediction of treatment discontinuation and recovery from Borderline Personality Disorder: Results from an RCT comparing Schema Therapy and Transference Focused Psychotherapy. Behav Res Ther. 2015;74:60–71. 26432172 10.1016/j.brat.2015.09.002

[61] First MB, Gibbon M, Spitzer RL, Williams JBW, Benjamin LS. Structured Clinical Interview for DSM-IV Axis II Personality Disorders (SCID-II). Washington, DC: American Psychiatric Press, Inc.; 1997.

[62] First MB, Szádóczky E, Unoka Z, Rózsa S. SCID-II: strukturált klinikai interjú a DSM-IV II-es tengelyén található személyiségzavarok felmérésére. OS Hungary Tesztfejlesztő; 2004.

[63] Viechtbauer W, Smits L, Kotz D, Budé L, Spigt M, Serroyen J, et al. A simple formula for the calculation of sample size in pilot studies. J Clin Epidemiol. 2015;68(11):1375–9. 26146089 10.1016/j.jclinepi.2015.04.014

[64] Rosenberg M. Society and the adolescent self-image. Princeton University Press, Princeton, N.J.; 1965.

[65] Sallay V, Martos T, Földvári M, Szabó T, Ittzés A. A Rosenberg Önértékelés Skála (RSES-H): alternatív fordítás, strukturális invariancia és validitás [Hungarian version of the Rosenberg Self-esteem Scale (RSES-H): An alternative translation, structural invariance, and validity]. Mentálhigiéné es Pszichoszomatika. 2014;15:259–75.

[66] Perczel-Forintos D, Ajtay G, Barna C, Kiss Z, Komlósi. S. Kérdőívek, becslőskálák a klinikai pszichológiában. 4 ed. Budapest, Hungary: Semmelweis Kiadó; 2019.

[67] Rózsa S, Szádóczky E, Füredi J. Psychometric properties of the Hungarian version of the shortened Beck Depression Inventory. Psychiatria Hungarica. 2001;16:384–402.

[68] Beck AT, Ward CH, Mendelson M, Mock J, Erbaugh J. An inventory for measuring depression. Arch Gen Psychiatry. 1961;4:561–71. 13688369 10.1001/archpsyc.1961.01710120031004

[69] Beck AT, Weissman A, Lester D, Trexler L. The measurement of pessimism: The Hopelessness Scale. J Consult Clin Psychol. 1974;42(6):861–5. 4436473 10.1037/h0037562

[70] Perczel-Forintos D, Sallai J, Rózsa S. Adaptation of the Beck Hopelessness Scale in Hungary. Psihologijske Teme. 2010;19(2):307–21.

[71] Steinberg L, Sharp C, Stanford MS, Tharp AT. New tricks for an old measure: the development of the Barratt Impulsiveness Scale-Brief (BIS-Brief). Psychol Assess. 2013;25(1):216–26. 23148649 10.1037/a0030550

[72] Horváthné Pató I, Szekeres T, Kresznerits S, Perczel-Forintos D. [The Barratt Impulsiveness Scale-Brief-8 in an Incarcerated Sample: Suicide Risk, Impulsivity and Mindfulness]. Psychiatr Hung. 2023;38(3):203–17. 37982268

[73] Carlson EB, Putnam FW. An update on the dissociative experiences scale. Dissociation: progress in the dissociative disorders. 1993.

[74] Kocsis-Bogár K. A szkizofrénia spektrum és a traumatikus életesemények összefüggései. Budapest: Semmelweis University; 2016.

[75] Garnefski N, Kraaij V. The cognitive emotion regulation questionnaire: Psychometric features and prospective relationships with depression and anxiety in adults. Eur J Psychol Assess. 2007;23(3):141–9.

[76] Geisler FCM, Vennewald N, Kubiak T, Weber H. The impact of heart rate variability on subjective well-being is mediated by emotion regulation. Pers Indiv Differ. 2010;49(7):723–8.

[77] Miklósi M, Martos T, Kocsis-Bogár K, Perczel Forintos D. A Kognitív Érzelem-Reguláció Kérdőív magyar változatának pszichometriai jellemzôi. Psychiatria Hungarica. 2011;26(2):102–11. 21653995

[78] Neff KD. The development and validation of a scale to measure self-compassion. Self Identity. 2003;2:223–50.

[79] Sági A, Köteles F, Komlósi V. Az Önmagunk Iránt Érzett Együttérzés (Önegyüttérzés) skála magyar változatának pszichometriai jellemzői. Pszichológia. 2013;33:293–312.

[80] Nunnally JC, Bernstein IH. Psychometric Theory (3rd Edition): New York: McGraw-Hill; 1994.

[81] Balsis S, Loehle-Conger E, Busch AJ, Ungredda T, Oltmanns TF. Self and informant report across the borderline personality disorder spectrum. Personal Disord. 2018;9(5):429–36. 28857585 10.1037/per0000259 PMC6082732

[82] Winter D, Herbert C, Koplin K, Schmahl C, Bohus M, Lis S. Negative evaluation bias for positive self-referential information in borderline personality disorder. PLoS ONE. 2015;10(1):e0117083. 25612212 10.1371/journal.pone.0117083 PMC4303263

