# Supplementary material for: Evaluating a brief MBCT programme for non-suicidal self-injury in individuals with BPD: a within-subject pre–post pilot study
Source: Borderline Personal Disord Emot Dysregul. 2026 Mar 9;13:10. doi: 10.1186/s40479-026-00337-3 (PMC13085490; doi:10.1186/s40479-026-00337-3)
Supplement: Supplementary file 1 — Supplementary Material 1 [file 40479_2026_337_MOESM1_ESM.pdf]

Supplementary Material S1. NSSI interview guide (standardised prompts and coding rules)

Purpose
This semi-structured interview assesses non-suicidal self-injury (NSSI) presence, methods (types), and frequency using standardised prompts and pre-specified coding rules. The interview was administered at baseline (T1) and post-intervention (T3) by trained clinical psychologists, using a consistent question order and coding procedures across groups and assessment points. The guide is provided to support methodological transparency and replicability.

Context
At T1, the NSSI block was embedded in a pre-class motivational interview format derived from the MBCT pre-class interview framework (Williams et al., 2015) and adapted to the study's BPD/NSSI focus and the team's cognitive–emotional reactivity formulation (Kresznerits et al., 2023, 2026). At T3, the interview focused on the NSSI block.

Administration
Administrators: Trained clinical psychologists.
Timing:

- T1 (baseline; 8–12 weeks pre-intervention): Motivational interview + NSSI interview, assess NSSI during the past 6 months
- T3 (post-intervention): NSSI interview (and brief safety check if clinically indicated), assess NSSI since the start of the MBCT-NSSI group (i.e., during the 9-week programme period).

Important note for interpretation: these reference windows differ (6 months vs. 9 weeks), which limits direct comparability of raw frequency across time points (also stated in the manuscript).

Interview format and duration

- Semi-structured: follow prompts in order; use optional probes as needed.
- Typical duration: ~10–20 minutes for the NSSI block (~45–60 minutes at T1 due to motivational interview context).

Safety and escalation (brief)
If the participant reports any suicidal intent, recent suicide attempt, or acute risk indicators during the conversation, pause the NSSI block and follow local clinical safety procedures (e.g., immediate clinical assessment, psychiatrist contact, emergency steps as per site protocol).

Part A. Pre-class motivational and safety interview (T1) (core points)

A1. Opening and alliance-building (rapport)
A2. "What brings you here?" Understanding BPD and NSSI in the individual (adapted cognitive–emotional reactivity model)
A3. Suicide risk and safety screening (motivational interview style, suicide prevention contract – suicidal ideation was not systematically assessed and was not analysed)
A4. "How will MBCT be helpful to you?" (personalised rationale + psychoeducation)
A5. Commitment and feasibility (attendance + home practice)

A6. Anticipated challenges (normalisation + planning)
A7. Confidentiality, safety agreement, and practical arrangements

Part B. NSSI block: scripts and prompts
B1. Definition and boundary-setting (script)

"In the next questions, I will ask about ***non-suicidal self-injury****. By this, we mean deliberately harming your body (especially your body tissue) on purpose without the intention to die, for example, cutting or hitting yourself. If something was done with the intention to die, we consider that* ***suicidal behaviour*** *and we will address it separately for safety reasons."*

Comprehension check: *"Does this definition make sense? When I say self-injury here, I mean without intent to die."*

B2. Screening for lifetime NSSI

1. **Lifetime presence**
   "Have you ever deliberately harmed yourself without trying to kill yourself?"
   - If **No** → code "No lifetime NSSI" and stop the block (typically does not occur due to eligibility criteria).
   - If **Yes** → continue.
2. **Reference-window presence**
   - T1: *"Have you done this in the past 6 months?"*
   - T3: *"Have you done this since the start of the MBCT-NSSI group?"*

Any self-injurious act endorsed with intent to die was not coded as NSSI and prompted the safety procedure

B3. NSSI methods (types): standard list + systematic probing

"What kinds of self-injury have you done?"
If the participant is uncertain, unfamiliar with terminology, or gives a vague response, provide examples and then probe systematically.

Standard method list (used for probing, queried systematically even if not endorsed spontaneously):

1. Cutting (e.g., with blade/knife)
2. Self-hitting / punching self
3. Scratching / carving / picking skin to cause injury
4. Burning (e.g., cigarettes, lighter, hot object)
5. Biting
6. Head-banging / hitting body against objects
7. Interfering with wound healing (reopening wounds)
8. Inserting objects under skin or nails
9. Self-tattooing
10. Other deliberate self-injury (specify):

For each endorsed method, ask:

- "Was this done on purpose to harm yourself?"
- "At the time, was there any intention to die?"

(If any suicidal intent is endorsed → trigger safety procedure; do not code as NSSI.)

B4. Frequency assessment

- T1: *"Thinking about the past 6 months, how many times or how often did you harm yourself without intending to die?"*
- T3: *"Thinking about since the start of the group, how many times or how often did you harm yourself without intending to die?"*

Anchoring probes if needed (optional):

- "Would you say it was closer to: a few times, monthly, weekly, or daily?"
- "Was it more or less than once per month?"
- "Did it happen in bursts (clusters) or evenly over time?"

Frequency response categories shown to the interviewer (do not show as a "test")
Assign the participant to one category using the rules below (Coding rules).

B5. Closing (brief)

"Thank you. Before we finish: is there anything important about your self-injury over this period that you think we should know for safety or support?"
(If clinical concern emerges → follow safety procedure.)

Coding rules (pre-specified)

- Core variables to code at each assessment point
  1. NSSI presence in the reference window (Yes/No)
  2. NSSI frequency category (ordinal; see frequency category coding)
  3. NSSI methods endorsed (checklist; see B3)
  4. Number of methods (count of endorsed method categories)

  Coding note: the coding is based on interview content and does not influence eligibility decisions during the interview.

- Frequency category coding (primary coding rule)
  Assign exactly one category for NSSI frequency in the reference period:
  - T1 baseline: past 6 months
    - 1 = several times a year (approximately 1–3 times/year)
    - 2 = approximately monthly (more than ~10 times/year; roughly monthly)
    - 3 = approximately weekly
    - 4 = daily or more frequent
  - T3 post-intervention: since group start
    - 0 = no self-injury since the start of the group
    - 1 = several times a year
    - 2 = approximately monthly
    - 3 = approximately weekly
    - 4 = daily or more frequent

  Rule for category 0 at T3:
  Use 0 only if the participant explicitly reports no NSSI episodes since the start of the group. Rule for uncertainty: If the participant cannot give a number, use anchoring probes and assign the closest category based on their best estimate and the interviewer's judgment. Document brief rationale in notes (e.g., "participant reported 2–3 episodes total → category 1").
  Frequency categories were assigned by the interviewer using standard prompts and anchoring probes.

Supplementary Material S2. Transparent Reporting of Evaluations with Nonrandomized Designs (TREND) 2024 checklist

Table S2. TREND Statement Checklist

Paper Section/ Topic	Item No	Descriptor	Reported? ✓	Section (s) Pg #
**Title and Abstract**				
Title and Abstract	1	• Information on how unit were allocated to interventions	✓	s2.1
		• Structured abstract recommended	✓	2#
		• Information on target population or study sample	✓	s2.1
**Introduction**				
Background	2	• Scientific background and explanation of rationale	✓	s1
		• Theories used in designing behavioral interventions	✓	s1
**Methods**				
Participants	3	• Eligibility criteria for participants, including criteria at different levels in recruitment/sampling plan (e.g., cities, clinics, subjects)	✓	s2.1 s2.2
		• Method of recruitment (e.g., referral, self-selection), including the sampling method if a systematic sampling plan was implemented	✓	s2.1 s2.2
		• Recruitment setting	✓	s2.1
		• Settings and locations where the data were collected	✓	s2.1
Interventions	4	• Details of the interventions intended for each study condition and how and when they were actually administered, specifically including:	✓	s2.3
		○ Content: what was given?	✓	s2.3
		○ Delivery method: how was the content given?	✓	s2.3
		○ Unit of delivery: how were the subjects grouped during delivery?	✓	s2.3
		○ Deliverer: who delivered the intervention?	✓	s2.3
		○ Setting: where was the intervention delivered?	✓	s2.3
		○ Exposure quantity and duration: how many sessions or episodes or events were intended to be delivered? How long were they intended to last?	✓	s2.3
		○ Time span: how long was it intended to take to deliver the intervention to each unit?	✓	s2.3
		○ Activities to increase compliance or adherence (e.g., incentives)	✓	s2.3
Objectives	5	• Specific objectives and hypotheses	✓	s1.1
Outcomes	6	• Clearly defined primary and secondary outcome measures	✓	s2.5
		• Methods used to collect data and any methods used to enhance the quality of measurements	✓	s2.5
		• Information on validated instruments such as psychometric and biometric properties	✓	s2.4 s3.1
Sample Size	7	• How sample size was determined and, when applicable, explanation of any interim analyses and stopping rules	✓	s2.1
Assignment Method	8	• Unit of assignment (the unit being assigned to study condition, e.g., individual, group, community)	✓	s2.2
		• Method used to assign units to study conditions, including details of any restriction (e.g., blocking, stratification, minimization)	✓	s2.2
		• Inclusion of aspects employed to help minimize potential bias induced due to non-randomization (e.g., matching)	✓	s2.2

Blinding (masking)	9	• Whether or not participants, those administering the interventions, and those assessing the outcomes were blinded to study condition assignment; if so, statement regarding how the blinding was accomplished and how it was assessed.	✓	s4.6
Unit of Analysis	10	• Description of the smallest unit that is being analyzed to assess intervention effects (e.g., individual, group, or community)	✓	s2.1
		• If the unit of analysis differs from the unit of assignment, the analytical method used to account for this (e.g., adjusting the standard error estimates by the design effect or using multilevel analysis)	--	
Statistical Methods	11	• Statistical methods used to compare study groups for primary methods outcome(s), including complex methods of correlated data	✓	s2.5
		• Statistical methods used for additional analyses, such as a subgroup analyses and adjusted analysis	✓	s2.5
		• Methods for imputing missing data, if used	✓	s2.5
		• Statistical software or programs used	✓	s2.5
**Results**				
Participant flow	12	• Flow of participants through each stage of the study: enrollment, assignment, allocation, and intervention exposure, follow-up, analysis (a diagram is strongly recommended)	✓	s2.2 Fig.1.
		o Enrollment: the numbers of participants screened for eligibility, found to be eligible or not eligible, declined to be enrolled, and enrolled in the study	✓	s2.2 Fig.1.
		o Assignment: the numbers of participants assigned to a study condition	✓	s2.2 Fig.1.
		o Allocation and intervention exposure: the number of participants assigned to each study condition and the number of participants who received each intervention	✓	s2.2 Fig.1.
		o Follow-up: the number of participants who completed the follow-up or did not complete the follow-up (i.e., lost to follow-up), by study condition	✓	s2.2 Fig.1.
		o Analysis: the number of participants included in or excluded from the main analysis, by study condition	✓	s2.2 Fig.1.
		• Description of protocol deviations from study as planned, along with reasons	✓	s4.5
Recruitment	13	• Dates defining the periods of recruitment and follow-up	✓	s2.2
Baseline Data	14	• Baseline demographic and clinical characteristics of participants in each study condition	✓	s3.1
		• Baseline characteristics for each study condition relevant to specific disease prevention research	✓	s3.1
		• Baseline comparisons of those lost to follow-up and those retained, overall and by study condition	✓	s3.3
		• Comparison between study population at baseline and target population of interest	✓	s4.5
Baseline equivalence	15	• Data on study group equivalence at baseline and statistical methods used to control for baseline differences	✓	s2.2 s3.2.2
